# Simultaneous bilateral hemorrhagic pleural effusion and hemorrhagic ascites during percutaneous nephrolithotomy: a case report

**DOI:** 10.3389/fmed.2026.1895920

**Published:** 2026-06-24

**Authors:** Haobin Peng, Jianxin Ou, Weilin Huang, Jiajun Wen, Ying Jiang, Xianping Wu, Manli Chen

**Affiliations:** Department of Anesthesiology, Shunde Traditional Chinese Medicine Hospital, Guangzhou University of Traditional Chinese Medicine, Foshan, Guangdong, China

**Keywords:** hemorrhagic peritoneal effusion, hemorrhagic pleural effusion, percutaneous nephrolithotomy, perioperative ultrasound, pleuroperitoneal communication

## Abstract

**Background:**

Percutaneous nephrolithotomy (PCNL) may cause severe thoracic and abdominal complications. Because their onset can be insidious and their intraoperative manifestations atypical, recognition may be delayed. We report a case of sudden bilateral hemorrhagic pleural effusion with hemorrhagic ascites during PCNL and discuss strategies for intraoperative identification and management.

**Case presentation:**

A 63-year-old woman underwent PCNL for multiple right renal calculi. During the procedure, renal pelvic bleeding occurred, followed by a sudden increase in airway pressure to 40 cmH2O, a decrease in oxygen saturation to 92–95%, and a fall in blood pressure to 78/59 mmHg. Bedside ultrasound revealed substantial bilateral pleural effusion and moderate intraperitoneal fluid accumulation. Emergency bilateral chest tube drainage and abdominal paracentesis were performed, evacuating a large volume of pale red blood-tinged fluid. After volume expansion, blood transfusion, and vasopressor support, the patient’s vital signs stabilized. She was discharged after 10 days of recovery, and the 3-month follow-up showed no significant abnormalities.

**Conclusion:**

During PCNL, sudden hypoxemia and elevated airway pressure should prompt immediate assessment for severe pleural or peritoneal effusion. In this case, the findings may have resulted from pressure-related communication between the abdominal and thoracic cavities, with fluid translocation through small diaphragmatic defects into both pleural spaces. Bedside ultrasound can serve as a first-line screening tool during PCNL. Continuous ultrasound monitoring, including wearable devices, may offer advantages in prone-position surgery, particularly when ongoing surveillance for pulmonary complications is required.

## Introduction

Percutaneous nephrolithotomy (PCNL) is considered the gold-standard surgical procedure for complex kidney stones (1). Michel et al. reported that complications associated with PCNL include hemorrhage, the need for transfusion, fever, and pleural injury (2). Lojanapiwat et al. reported that the incidence of pleural complications during supracostal PCNL may be as high as 15.3% (3). At present, middle-pole access is commonly used for PCNL because it can effectively address stones in the renal pelvis and most upper and lower calyceal stones, while providing broad applicability and relatively favorable safety (4). However, pleural effusion may still occur even with a subcostal approach and in the absence of direct pleural injury. This may be related to the need for continuous high-pressure saline irrigation during PCNL (5). Because irrigation fluid may mix with blood during PCNL, the risk of hemorrhagic pleural effusion or hemorrhagic ascites may increase and may persist for an extended period (6). Therefore, early intraoperative identification of related complications is clinically important.

Early recognition of thoracoabdominal effusion during PCNL can be challenging. Auscultation is limited in the prone position, and symptoms may be masked by positive-pressure ventilation. These factors can make the condition difficult to detect and may pose a serious threat to patient safety, while also challenging the judgment and management capacity of the anesthesiologist. This case report describes bilateral bloody thoracoabdominal effusion during PCNL and discusses the importance of early recognition and potential management strategies.

A review of the English-language literature revealed no previously reported cases of simultaneous hemorrhagic effusion involving both bilateral pleural cavities and the abdominal cavity during PCNL, suggesting that this occurrence is extremely rare. Most reported PCNL-related thoracoabdominal complications involve a single cavity. Hua et al. reported a case of shock caused by unilateral bloody pleural effusion due to diaphragmatic injury during PCNL (7). Sinha et al. documented a case of markedly delayed unilateral massive hemothorax that occurred 10 days after PCNL (8). Pérez-Palenzuela et al. reported a rare case of urinothorax after PCNL (9). Sharma et al. reported intraoperative fluid extravasation during PCNL with accumulation in the abdominal cavity (Morison’s pouch), without thoracic involvement (10). Kazemi et al. described acute liver injury and contralateral pleural effusion following PCNL (11). To the best of our knowledge, this case represents one of the few reported occurrences of simultaneous bilateral hemorrhagic pleural effusion and hemorrhagic ascites during PCNL.

## Case report

Before drafting this report, written informed consent was obtained from the patient.

### General condition and preoperative preparation

The patient was a 63-year-old woman who was admitted on October 22, 2025, because she was 2 months post-radiotherapy for nasopharyngeal carcinoma. She had a history of hypertension. In July 2025, she had been diagnosed with nasal cavity squamous cell carcinoma (T1N0M0, stage I) and completed intensity-modulated radiotherapy (IMRT) in August 2025. During this admission for follow-up evaluation, abdominal CT revealed multiple right renal calculi with hydronephrosis. Surgical intervention was indicated, and PCNL was planned. The preoperative assessment classified the patient as ASA physical status II. Her preoperative complete blood count showed a hemoglobin level of 113 g/L, and coagulation function was normal. Cefazolin was administered as antibiotic prophylaxis for 3 days before surgery.

### Intraoperative course

**Figure 1:**
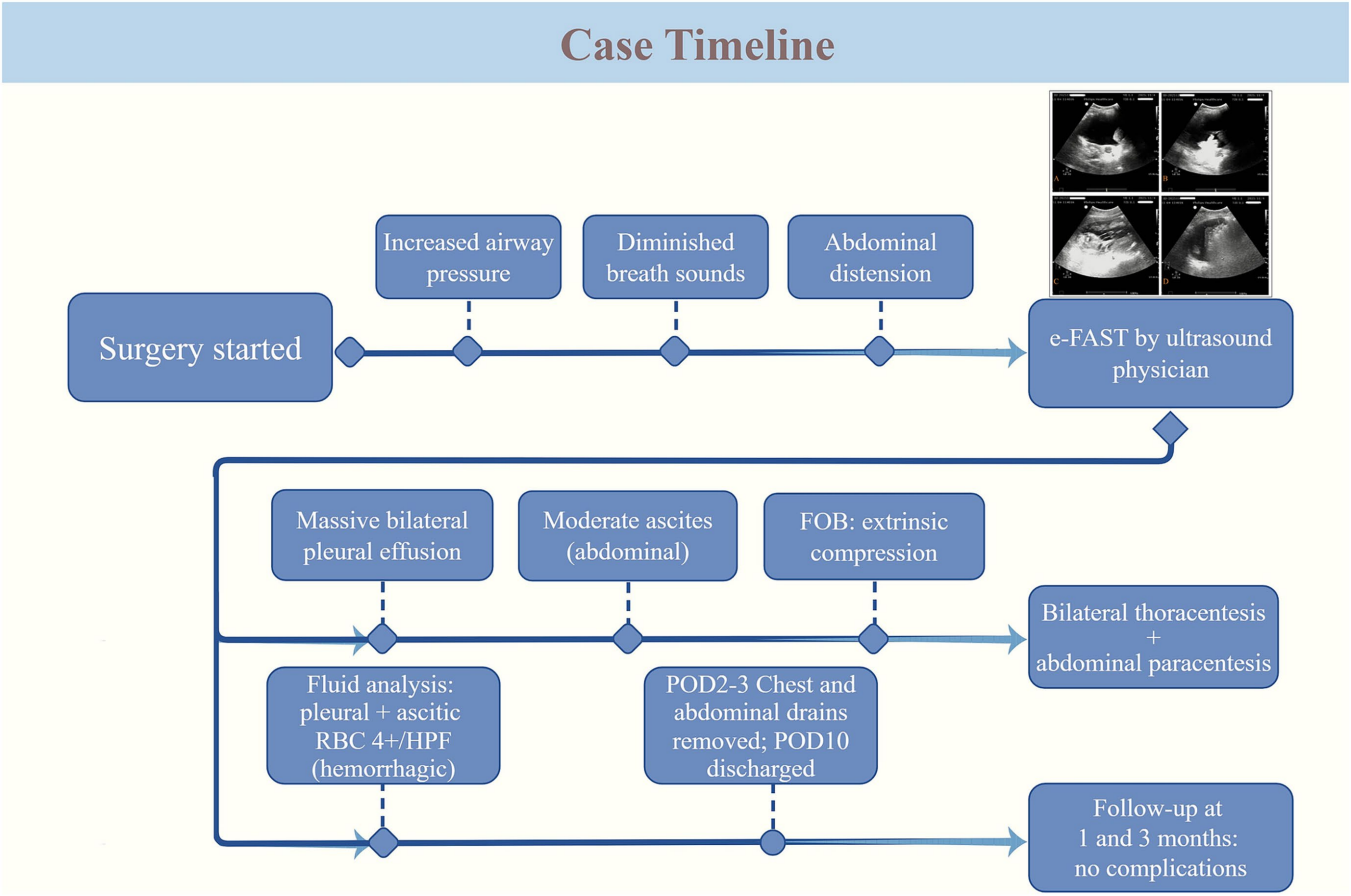
Case timeline. Key events from surgery started through follow-up, created by Figdraw. e-FAST, extended focused assessment with sonography for trauma; FOB, fiberoptic bronchoscopy; HPF, high power field; POD, postoperative day.

General anesthesia was induced with endotracheal intubation, and the key events are summarized in a timeline (Figure 1). The operation was performed with the patient in the prone position. Under ultrasound guidance, the upper calyx of the right kidney was successfully punctured at the junction of the 11th intercostal space and the posterior axillary line. EMS pneumatic ballistic lithotripsy was used during the procedure. Approximately 1.5 h after the start of surgery, the anesthesiologist observed a rapid increase in airway pressure to 40 cmH2O (baseline, 20–25 cmH2O), a decrease in oxygen saturation to 92–95%, with a minimum of 87%, a fall in blood pressure to 78/59 mmHg, and a heart rate of 132 beats/min.

**Figure 2:**
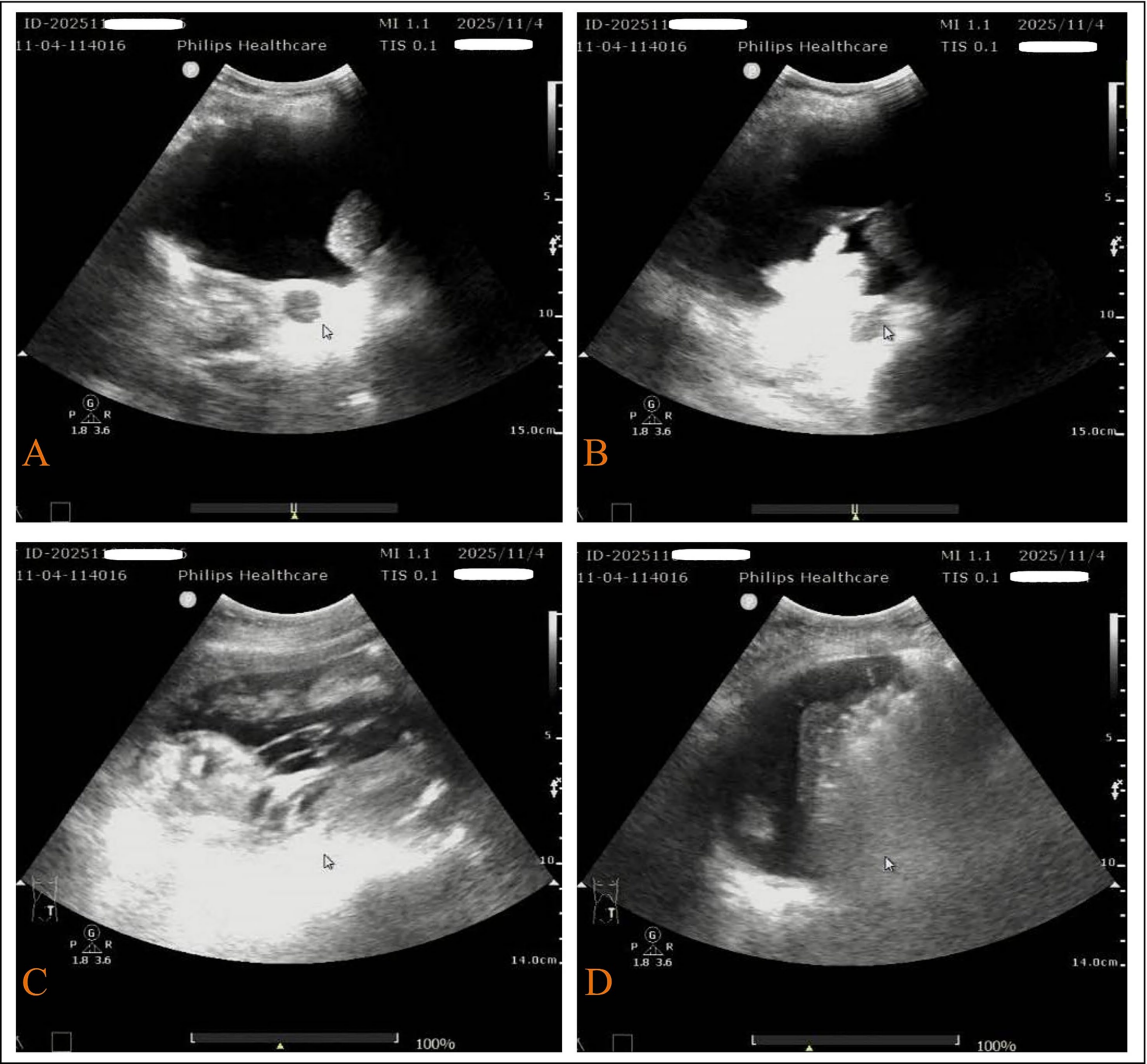
Intraoperative e-FAST imaging of pleuroperitoneal effusion. **(A)** Left chest: large pleural effusion. **(B)** Right chest: large pleural effusion. **(C)** Right perinephric region: fluid accumulation with nephrostomy tube visualized. **(D)** Abdomen: moderate ascites. e-FAST, extended focused assessment with sonography for trauma. All image captures preserved original metadata including essential information and dates, while patient names and institutional details were redacted in compliance with privacy protection protocols.

At that time, lung auscultation revealed markedly diminished bilateral breath sounds, raising concern for an acute thoracic complication. The surgical team was immediately asked to suture the wound, and the patient was turned to the supine position, which took approximately 3 min. The abdomen was markedly distended. Emergency bedside ultrasound demonstrated large bilateral pleural effusions and moderate abdominal effusion, as shown in Figure 2. Fiberoptic bronchoscopy revealed no foreign body in the trachea, but the secondary bronchi showed extrinsic narrowing, as shown in Supplementary Video 1. Immediate bilateral thoracentesis/chest drainage and abdominal paracentesis were performed, draining a large volume of light red hemorrhagic fluid. At the same time, central venous access was established for volume resuscitation and blood transfusion. Norepinephrine was infused, and acidosis was corrected with calcium supplementation as needed. Immediate arterial blood gas analysis showed pH 7.00, PaCO2 72.9 mmHg, base excess −13 mmol/L, Ca^2^ + 1.15 mmol/L, and hemoglobin 82 g/L. One hour after surgery, hemoglobin was reassessed at 58 g/L. After transfusion of 4 units of packed red blood cells, the patient’s vital signs stabilized (blood pressure, 152/90 mmHg; SpO2, 100%), and she was transferred to the intensive care unit.

### Postoperative follow-up

**Figure 3:**
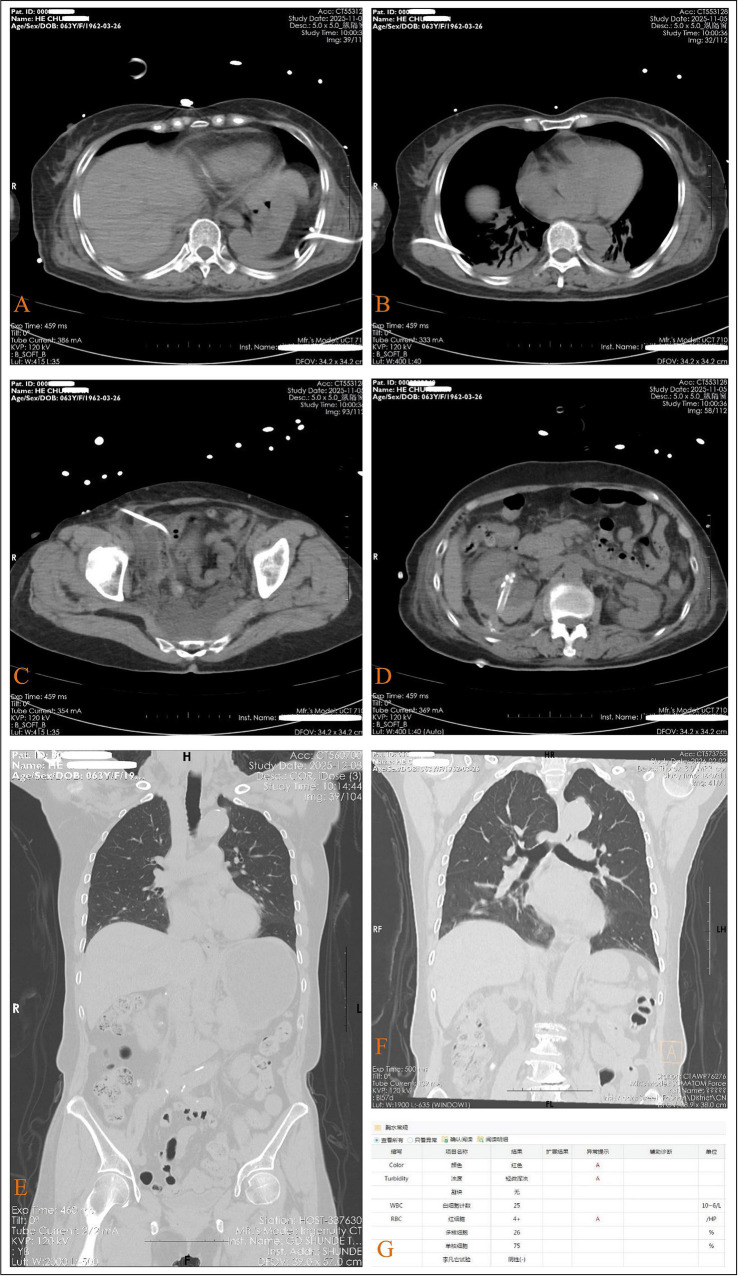
Postoperative chest and abdominal drains, follow-up CT imaging, and hemorrhagic pleural fluid analysis. **(A)** Left chest: chest tube *in situ*. **(B)** Right chest: chest tube in situ. **(C)** Abdomen: abdominal drain in situ. **(D)** Right kidney: nephrostomy tube in place. **(E)** One-month follow-up thoracoabdominal CT: no abnormalities. **(F)** Three-month follow-up thoracoabdominal CT: unremarkable. **(G)** Postoperative pleural fluid analysis: red blood cell count 4 + per high power field. All image captures preserved original metadata including essential information and dates, while patient names and institutional details were redacted in compliance with privacy protection protocols.

On postoperative day 1, the tracheal tube was removed. Postoperative CT showed a hematoma in the right renal pelvis and subcapsular region, fluid accumulation in the abdominal and pelvic cavities, and mild exudation in both lungs. Routine analysis of peritoneal and pleural effusion showed red fluid with a red blood cell count of 4 per high-power field, as shown in Figure 3G. On postoperative day 2, the patient was transferred back to the urology department. She was discharged on postoperative day 10 with a right renal stoma catheter in place. Follow-up assessments were performed at 1 month and 3 months after surgery, and chest CT showed no significant abnormalities, as illustrated in Figure 3.

## Discussion

Acute intraoperative hypoxemia and elevated airway pressure during PCNL should raise concern for acute massive hemorrhagic pleural effusion or hemorrhagic ascites. When sudden hypoxemia occurs during surgery, anesthesiologists should differentiate among several potential causes, including pneumothorax, endobronchial intubation, pulmonary edema, pulmonary embolism, and bronchospasm (12). This case suggests that substantial hemorrhagic pleural and peritoneal effusion should also be considered as important differential diagnoses, particularly during PCNL. Initial clinical clues include a sharp increase in airway pressure, reduced bilateral breath sounds, and obvious abdominal distension during intraoperative monitoring and physical examination (13). When these findings occur together, considerable pleural and abdominal effusion should be strongly suspected. At this point, bedside radiography or ultrasound using the eFAST approach can rapidly identify thoracic and abdominal pathology (10). Although CT can provide more precise etiological information, such as the location of diaphragmatic defects and the extent of effusion, it is time-consuming and carries transport-related risk, making it unsuitable for rapid intraoperative emergency management (14). Closed chest drainage is the initial treatment for hemorrhagic pleural effusion. When clinical suspicion is strong, treatment should not be delayed while awaiting imaging confirmation, because delay may compromise lifesaving intervention (15). Overall, rapid recognition of clinical signs remains an essential skill for anesthesiologists.

Bedside ultrasound should be considered a primary screening tool during PCNL, and continuous ultrasound monitoring has considerable potential for future application. In this case, routine auscultation was restricted because the patient was in the prone position and covered with sterile drapes. Rapid bedside ultrasound enabled diagnosis and guided emergency drainage within minutes. Xie et al. reported that lung ultrasound had diagnostic performance comparable to CT for postoperative hypoxemia caused by pleural pathology, with a sensitivity of 92.9%, specificity of 96.0%, and diagnostic accuracy of 95.1% for pleural effusion (16). Kirkpatrick et al. also validated the high diagnostic performance of e-FAST (17). However, an important implication of this case is that on-demand ultrasound examination during PCNL may still be associated with delay. Although the interval from increased airway pressure to ultrasound confirmation and drainage was short in this case, oxygen saturation continued to decline during this period, and hemoglobin decreased from 82 g/L to 58 g/L. If continuous ultrasound monitoring could be implemented during surgery, the trend of fluid accumulation might be detected earlier, enabling true early warning rather than only rescue intervention.

Wearable color Doppler ultrasound devices have been successfully applied for continuous cardiac monitoring and in perioperative settings, including in pregnant women and neonates (18–21). These technologies may be theoretically advantageous for procedures performed in the prone position under sterile draping, such as PCNL, where continuous operation of conventional ultrasound is difficult. However, their use for detecting pleural or peritoneal effusion remains investigational and is not directly supported by the present case. Instead, this case reinforces the established role of bedside e-FAST ultrasound as a rapid, reliable, and immediately available diagnostic tool for intraoperative thoracoabdominal complications.

Clinicians should remain alert to the possibility that communication between the thoracic and abdominal cavities may open under high-pressure conditions, leading to severe complications in both cavities. In this case, intraoperative ultrasound and postoperative CT showed no pneumothorax, but substantial hemorrhagic effusion was present in both the thoracic and abdominal cavities. These observations indicate the absence of direct pleural breach. A possible explanation for these findings is as follows. During PCNL, renal pelvic bleeding, accidental perforation, or excessive irrigation pressure may increase retroperitoneal pressure, allowing irrigation fluid to enter the abdominal cavity directly or indirectly (22). After intra-abdominal pressure increases, lavage fluid may translocate upward into both pleural cavities through congenital or iatrogenic diaphragmatic defects (23). Of note, intra-abdominal fluid can cross the diaphragm via inherent holes or lymphatic vessels, driven by the abdominothoracic pressure gradient (24). Xu et al. found a 5.6% incidence of pleural effusion after PCNL even without direct pleural injury (25). Similarly, laparoscopic CO₂ pneumothorax occurs within minutes through diaphragmatic defects (13, 26). By analogy, the high irrigation pressure during PCNL can force hemorrhagic fluid through the same defects, causing rapid bilateral effusion. Notably, after the patient was turned from prone to supine, ultrasound showed only moderate ascites despite massive bilateral pleural effusion. This disparity suggests that hemorrhagic fluid may have translocated through microscopic diaphragmatic defects into the chest during the prone position, driven by high irrigation pressure and abdominal compression (27, 28). Thus, porous diaphragm syndrome represents a plausible but speculative mechanistic explanation for the observed pleuroperitoneal fluid translocation in this case. Once abdominal effusion is detected intraoperatively during PCNL, clinicians should remain highly vigilant for possible extension into the thoracic cavity.

This case has several important limitations. First, intraoperative biochemical analysis of the drainage fluid, including creatinine, urea nitrogen, and protein levels, was not performed. Therefore, the relative proportions of irrigation fluid and blood could not be accurately determined, and urinary effusion could not be excluded. Second, the use of continuous ultrasound monitoring during PCNL is not yet supported by prospective studies. Third, fluoroscopic confirmation of a subcostal approach was not obtained because ultrasound guidance was used. However, the absence of pneumothorax and the presence of bilateral pleural effusion on intraoperative ultrasound and postoperative CT make direct pleural injury unlikely. Fourth, the patient had a history of radiotherapy for nasopharyngeal carcinoma, which could theoretically affect tissue healing and pleural integrity. However, preoperative chest CT showed no pleural or pulmonary abnormalities, and neither intraoperative nor postoperative findings suggested radiotherapy-related tissue fragility. We therefore considered that this history did not substantially influence the observed complications.

In summary, a sudden increase in airway pressure and a decrease in oxygen saturation during PCNL should promptly raise suspicion for substantial pleural or peritoneal effusion. Bedside ultrasound should be used as a primary screening tool during PCNL. Wearable cardiac ultrasound imaging devices may have promising applications for continuous monitoring in this surgical context.

## Data Availability

The original contributions presented in the study are included in the article/Supplementary material, further inquiries can be directed to the corresponding authors.

## Supplementary material

SUPPLEMENTARY VIDEO 1 Intraoperative emergency fiberoptic bronchoscopy shows no foreign body in the trachea; however, extrinsic compression of the right secondary bronchi is observed.
